# Depression with Comorbid Diabetes: What Evidence Exists for Treatments Using Traditional Chinese Medicine and Natural Products?

**DOI:** 10.3389/fphar.2020.596362

**Published:** 2021-01-25

**Authors:** Yanting Lu, Tao An, Hu Tian, Xueqin Gao, Furong Wang, Shijun Wang, Ke Ma

**Affiliations:** ^1^ Shandong Co-Innovation Center of Classic TCM Formula, Shandong University of Traditional Chinese Medicine, Jinan, China; ^2^ College of TCM, Shandong University of Traditional Chinese Medicine, Jinan, China; ^3^ School of Pharmaceutical Sciences, Qilu University of Technology (Shandong Academy of Sciences), Jinan, China

**Keywords:** depression, diabetes, comorbidity, natural products, TCM

## Abstract

Comorbidity between diabetes mellitus (DM) and depression, two chronic and devastating diseases spreading worldwide, has been confirmed by a large body of epidemiological and clinical studies. Due to the bidirectional relationship between DM and depression, this comorbidity leads to poorer outcomes in both conditions. Given the adverse effects and limited effectiveness of the existing therapies for depression associated with diabetes, the development of novel therapeutic drugs with more potency and fewer side effects is still the most important goal. Hence, many researchers have made great efforts to investigate the potential usefulness of traditional Chinese medicine (TCM) and natural products, including natural extracts and purified compounds, in the treatment of comorbid depression in diabetes. Here, we reviewed the related literature on TCM and natural products that can remedy the comorbidity of diabetes and depression and presented them on the basis of their mechanism of action, focusing on shared risk factors, including insulin resistance, oxidative stress and inflammation, and nervous disturbances. In short, this review suggests that TCM and natural products could expand the therapeutic alternatives to ameliorate the association between DM and depressive disorders.

## Introduction

Diabetes mellitus (DM) is a prevalent chronic metabolic disease characterized by high blood glucose levels, whereas depression is a mood disorder that is marked by a loss of interest, constant sadness, feelings of guilt or low self-worth, tiredness, disturbed sleep or appetite, and poor concentration (Koye et al., 2018; Lim et al., 2018; Lu et al., 2019). Statistics from the International Diabetes Federation (IDF) revealed that 451 million people lived with DM worldwide in 2017, and these figures are expected to reach 700 million by 2045 (Cho et al., 2018). Moreover, the accelerated pace of life and increased social pressure further lead to a fast-growing incidence of depression. The World Health Organization (WHO) projected that by the year 2030, diabetes would become the seventh leading cause of death worldwide and depression would rank first in global disease burdens (Lepine and Briley, 2011; Roshan and Stanton, 2013).

Considerable evidence supporting a close association between DM and depression has been published. On the one hand, pre-existing depression increases the risk of DM. As early as the 17th century, Thomas Willis, a famous British doctor and anatomist, hypothesized that long-term grief could sometimes cause diabetes. Strong epidemiological evidence provided by longitudinal studies has also indicated a high incidence of DM in individuals who previously developed depression (Knol et al., 2006; Engum, 2007; Golden et al., 2008). In terms of mechanism, the physiological changes elicited by depression (e.g., the release of counter-regulatory hormones, stimulation of sympathetic nervous system release, and altered inflammation state) can induce peripheral insulin resistance, which appears to be the main responsible for DM development (Champaneri et al., 2010). Moreover, poor behavioral factors associated with depression, such as lack of physical inactivity and high caloric intake, can partially contribute to the development of diabetes (Strine et al., 2008).

On the other hand, DM appears to increase the risk of incident or recurrent depression. For example, continuous hyperglycemia in diabetic patients can result in the activation of the hypothalamic-pituitary-adrenal (HPA) axis, which leads to endocrine disorders, characterized by the excess release of cortisol, a hormone that plays an important role in depression (Chiodini et al., 2006; Yi et al., 2010). In addition, several studies have suggested that diagnosed but not undiagnosed diabetes is significantly associated with depression, indicating that the burden of diabetes and/or the psychological stress experienced while coping with diabetes might relate to these depressive symptoms (Knol et al., 2007; Icks et al., 2008; Li et al., 2016). Likewise, epidemiologic studies have demonstrated that the incidence of depression in diabetic patients is two times higher than in the general population (Anderson et al., 2001). Although the relationship between DM and depression has been confirmed, compared with some widely studied complications associated with DM, including retinopathies and atherosclerosis, the comorbidity of diabetes and depression is greatly underestimated due to some circumstances, such as stealthiness, different syndrome types, and difficulties in the assessment of depression.

It should be noted that depression and diabetes each increases the risk of the other in a bidirectional interaction. A few systematic reviews and meta-analysis have estimated that depression accounts for a 60% increase in diabetes risk, and diabetes was estimated to account for a 24% increased risk of depression (Mezuk et al., 2008; Rubin et al., 2008; Yu et al., 2015; Khaledi et al., 2019; Nouwen et al., 2019). The comorbidity of DM and depression thus leads to higher medical care costs, worse prognosis, increased severity, decline in quality of life, and increased treatment resistance and mortality for both diseases (Pradeepa and Mohan, 2017).

**FIGURE 1:**
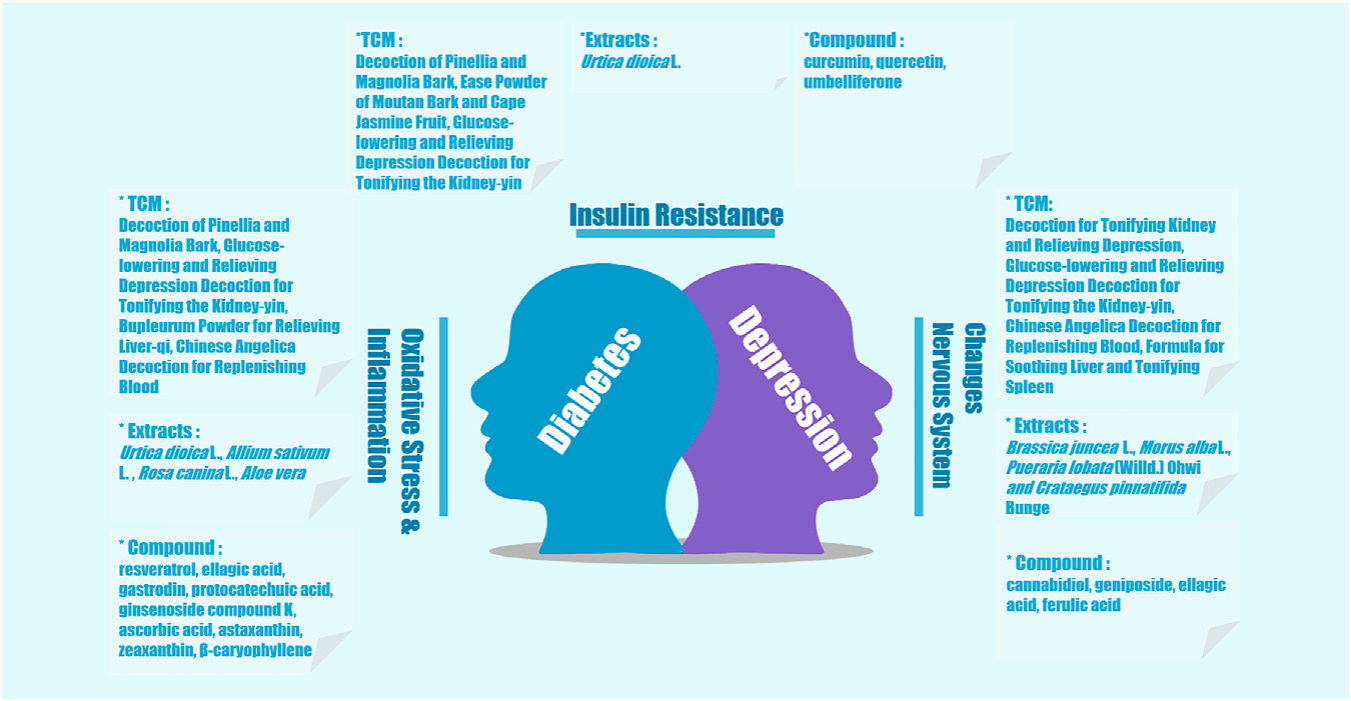
Schematic of TCM and natural products against comorbid depression with diabetes and their related mechanism.

In case of comorbid depression with diabetes, a key therapeutic aim is to relieve depression and improve blood glucose levels (Zhang and Feng, 2016). Clinically, it is generally considered that comorbid diabetes and depression should be treated with a combination of hypoglycemic drugs with antidepressants (Li et al., 2018). Although antidepressants are employed for pharmacological interventions in DM, their effectiveness in the improvement of depressive symptoms is moderate (Petrak et al., 2015). Apart from the documented adverse reactions like cardiometabolic effects, a large number of antidepressant agents, such as mirtazapine, can increase appetite in patients and result in a high risk of weight gain, which is not conducive to the control of blood sugar (Gill et al., 2020). Therefore, it is urgent to explore and discover novel agents with better efficacy and fewer adverse reactions. Given the multicomponent and multitarget characteristics of traditional Chinese medicine (TCM) as well as the value of natural products (natural extracts and single isolated compounds) for drug discovery, a growing body of research has investigated and established therapeutic effects and related mechanisms of multiple TCM interventions and natural products on diabetic depression (Zheng et al., 2020). We summarized these TCM and natural products and their effects against the comorbidity of diabetes and depression (Tables 1–3 and Figure 1) and classified them according to their mechanism of action, as outlined below.

**TABLE 1:** TCM formulae with effects on depression associated with diabetes.

TCM-preparation	Botanical drugs included	Species	Action	References
Decoction of Pinellia and Magnolia Bark	Pinelliae, officinal magnolia bark, Poria cocos, fresh ginger, Folium Perillae	*Pinellia ternate* (Thunb.) Makino*, Magnolia officinalis* Rehd. et Wils.*, Smilax glabra* Roxb.*, Perilla frutescens* (L.) Britt.*, Zingiber officinale* Rosc.	1) Suppresses NLRP3 inflammasome activation.	Jia et al. (2017)
			2) Improves peripheral insulin signaling impairment in the liver and brain in CUMS rats.	
Bupleurum Powder for Relieving Liver-qi	Pericarpium Citri Reticulatae, Radix Bupleuri, Rhizoma Ligustici Chuanxiong, Rhizoma Cyperi, Fructus Aurantii, Paeonia lactiflora, Radix Glycyrrhizae.	*Bupleurum chinense* DC.*, * *Citrus reticulata* Blanco*, Ligusticum striatum* DC.*, Cyperus rotundus* L.*, Citrus aurantium*L.*, Paeonia lactiflora* Pall*., Glycyrrhiza glabra* L.	1) Suppresses the TLR4/MyD88/NF-κB pathway and NLRP3 inflammasome activation, and improves insulin signaling in CUMS rats.	Jia et al. (2018)
Chinese Angelica Decoction for Replenishing Blood	Radix Angelicae Sinensis, Radix Astragali	*Angelica sinensis* (Oliv.) Dielss*, Astragalus mongholicus* Bunge	1) Inhibits systemic inflammatory response by reducing serum levels of inflammatory factors such as TNF-α, CRP, IL-6 and IL-8	Wang et al. (2018), Wang et al. (2020a), Wang et al. (2020b)
			2) Improves the depression-like behavior of Goto-Kakizaki rats, achieves hippocampal protection, and its antidepressant mechanism involves the CREB/BDNE/TrkB pathway.	
Ease Powder of Moutan Bark and Cape Jasmine Fruit	Radix Bupleuri, Radix Angelicae Sinensis, Paeonia lactiflora, Poria cocos, Rhizoma Atractylodis Macrocephalae, Radix Glycyrrhizae, Moutan Bark, Cape Jasmine	*Bupleurum chinense* DC.*, Angelica sinensis* (Oliv.) Dielss*, Paeonia lactiflora* Pall*, Smilax glabra* Roxb.*, Atractylodes lancea* (Thunb.) DC.*, Glycyrrhiza glabra* L.*, Paeonia suffruticosa* Andr.*, Gardenia jasminoides* Ellis	1) Regulates the IRS2-PI3K signaling pathway in liver tissues.	Wang et al. (2013), Zhang et al. (2017)
Formula for Soothing Liver and Tonifying Spleen	Radix Bupleuri, Poria cocos, Radix Astragali, Rhizoma Acori Tatarinowii, Radix Salviae Miltiorrhizae, Huai wheat	*Bupleurum chinense* DC.*, Smilax glabra* Roxb.*, Astragalus mongholicus* Bunge*,* *Acorus calamus var. angustatus* Besser*, Salvia miltiorrhiza* Bunge*, Triticum aestivum L.*	1) Upregulates the expression of BDNF mRNA and protein levels to improve the depressive symptoms associated with diabetes mellitus	Lei et al. (2017)
Decoction for Tonifying Kidney and Relieving Depression	Radix Rehmanniae Preparata, Fructus Corni, Fructus Schisandrae Chinensis, Radix Bupleuri, Radix Paeoniae Alba, Rhizoma Acori Tatarinowii, Semen Ziziphi Spinosae.	*Rehmannia* *glutinosa* (Gaertn.) DC.*, Cornus officinalis* Siebold & Zucc*., Schisandra chinensis* (Turcz.) Baill.*, Bupleurum chinense* DC.*, Paeonia lactiflora* Pall*., Acorus tatarinowii* Schott*,* *Ziziphus jujuba* Mill.	1) Decreases cortisol level and improves dysfunction of the HPA axis in patients with type 2 diabetes and depression.	Lu (2012)
Glucose-lowering and Relieving Depression Decoction for Tonifying the Kidney-yin	Radix Astragali, Radix Rehmanniae Preparata, Fructus Corni, Barbary Wolfberry, Semen Cuscutae, Cortex Eucommiae, Radix Salviae Miltiorrhizae, Cortex Moutan, Radix Achyranthis Bidentatae, Rhizoma Curcumae Longae, Fructus Forsythiae	*Astragalus mongholicus* Bunge*, Rehmannia glutinosa* (Gaertn.) DC.*, Cornus officinalis* Siebold & Zucc*., * *Lycium barbarum* L.*, Cuscuta chinensis* Lam*, Eucommia ulmoides* Oliv.*, * *Salvia miltiorrhiza* Bunge*, Paeonia suffruticosa* Andr.*, Achyrantes bidentata* Bl.*, Curcuma longa* L.*, Hypericum perforatum* L.	1) Improves insulin resistance.	Wang et al. (2014), Wang et al. (2015), Yang et al. (2018), Yang et al. (2019), Yang et al. (2020)
			2) Decreases the expression of IL-1β and TNF-α.	
			3) Reduces hippocampal CORT expression by inhibiting the expression of 11𝛽-HSD1 and increasing the levels of GR in the hippocampus.	

**TABLE 2:** Natural extracts with effects on depression associated with diabetes.

Medicinal plant	Active parts	Action	References
*Allium sativum* L.	Homogenate	1) Attenuates brain MDA levels and increases SOD and GPx activities.	Rahmani et al. (2020)
*Aloe vera*	Mucilaginous	1) Increases activities of hippocampal SOD and CAT and reduces MDA levels in the hippocampus tissue of STZ-induced diabetic rats	Tabatabaei et al. (2017)
*Brassica juncea* L.	Leave	1) Increases dopamine, norepinephrine, and serotonin in the brain.	Thakur et al. (2014)
*Morus alba* L.	Root bark	1) Restores BDNF levels in the prefrontal cortex through ERK and Akt signaling.	Ye et al. (2017)
*Pueraria lobata* (Willd.) Ohwi *and Crataegus pinnatifida* Bunge	Whole	1) Promotes BDNF expression and ERK activation to prevent depression in a diabetic rat model.	Luo et al. (2016)
*Rosa canina* L.	Fruits	1) Attenuates impairment of recognition memory and depressive-like behavior through the modulation of oxidative stress in the STZ model of diabetes in the mouse brain.	Farajpour et al. (2017)
*Urtica dioica* L.	Leaves	1) Improves hippocampal GLUT4 mRNA expression.	Patel and Udayabanu (2014); Patel et al. (2018)
		2) Upregulates BDNF, TrKB, Cyclin D1, Bcl2, and autophagy, downregulates iNOS mRNA expression in the hippocampus of diabetic mice, and decreases the expression of TNF-α in hippocampus of diabetic mice.	

**TABLE 3:** Natural compounds with effects on depression associated with diabetes.

Natural compounds	Plant source	Action	References
Ascorbic acid	*Citrus sinensis, Actinidia chinensis* Planch.	1) Ameliorates oxidative stress and inflammatory response by reducing catalase levels, increasing SOD content and CAT activity and IL-10 levels in the prefrontal cortex of diabetic rats.	Shivavedi et al. (2019)
Astaxanthin	*Haematococcus pluvialis, Oncorhynchus tschawytscha*	1) Downregulates the expression of IL-6, IL-1β, and COX-2 in the hippocampus.	Zhou et al. (2017)
β-Caryophyllene	*Syzygium aromaticum* (L.) Merr. and L.M. Perry, *Piper nigrum* L., *Salvia rosmarinus* Spenn.	1) Inhibits of inflammation/cytokines such as IL-1β, TNF-α, and IL-6	Aguilar-Avila et al. (2019)
Cannabidiol	*Cannabis sativa* L.	1) Activates the serotonergic system through 5-HT1A receptors	de Morais et al. (2018); Jesus et al. (2019)
Curcumin	*Curcuma longa* L.	1) Improves insulin sensitivity, upregulates the phosphorylation of IRS-1 and AKT, and inhibits GSK-3β, PEPCK and glucose 6-phosphatase.	Shen et al. (2017)
Ellagic acid	*Fragaia ananassa* (Duchesne ex Weston)	1) Modulates inflammation status and BDNF expression.	Farbood et al. (2019)
Gastrodin	*Gastrodia elata* Bl.	1) Inhibits ER stress and NLRP3 inflammasome activation in db/db mice.	Ye et al. (2018)
Geniposide	*Gardenia jasminoides* J.Ellis	1) Enhances BDNF expression in the hippocampus of streptozotocin-evoked diabetic mice	Wang et al. (2016)
Ginsenoside compound K	*Panax ginseng* C. A. Mey.	1) Inhibits ER stress and the NLRP3 inflammasome pathway	Li et al. (2020)
Protocatechuic acid	*Salvia miltiorrhiza* Bunge	1) Suppresses oxidative damage, inflammation, and the activities of caspase-3 and acetylcholinesterase in diabetic rats.	Adedara et al. (2019)
Quercetin	*Euonymus alatus* (Thunb.) Siebold, *Rosa canina L.*, *Allium cepa* L. et al.	1) Upregulates GLUT4 expression.	Anjaneyulu et al. (2003)
Resveratrol	*Polygonum cuspidatum* Sieb. and Zucc., *Paeonia lactiflora* Pall., *Vatica rassak* (Korth.) Blume	1) Inhibits oxidative stress.	Jing et al. (2013); Sahin et al. (2019)
		2) Reduces astrocytic activation as well as TNF-α and IL-6 transcripts in the hippocampus of diabetic rats.	
Umbelliferone	*Daucus carota* L.*, Coronilla varia* L.*, Ruta graveolens* L.	1) Attenuates CUMS-induced-insulin resistance in rats	Su et al. (2016)
Zeaxanthin	*Spinacia oleracea* L., *Brassica oleracea* L.	1) Reduces the excessive production of IL-6, IL-1β, and TNF-α	Zhou et al. (2018)

## TCM and Natural Products with Effects on Insulin Resistance

### Insulin Resistance and Comorbid Depression with Diabetes

Insulin resistance caused by the decrease in insulin sensitivity disrupts the entry of glucose into muscle, liver, and adipose tissue, which eventually leads to diabetes. Physiologically, the interaction of insulin with the insulin receptor (InsR) on the cell membrane activates an intrinsic tyrosine protein kinase, which autophosphorylates the receptor as well as downstream insulin receptor substrates (IRSs) (Jung and Choi, 2014). The phosphorylation of IRS proteins on tyrosine residues activates downstream PI3K-AKT signaling, which alters the activity or expression of several key factors involved in glucose metabolism and glycogen/lipid/protein synthesis, such as glycogen synthase kinase (GSK)-3*β*, regulatory element-binding protein-1c (SREBP1-c), and glucose transporter (GLUT) translocation, especially GLUT4 (Beale, 2013). The insulin-mediated IRS-PI3K-AKT signaling pathway is therefore a key pathway affecting the metabolic effects of insulin and is usually dysregulated in people with diabetes (Yaribeygi et al., 2019). Moreover, impairment of insulin signaling is also present in high-fat diet (HFD)-induced diabetic rats with depressive-like behavior (Papazoglou et al., 2015). Furthermore, a meta-analysis of 21 studies found a statistically significant association between depression and insulin resistance, and this positive association suggests a biological link between depression and DM (Kan et al., 2013). Insulin receptors are expressed throughout the brain, and several studies have indicated the ability of insulin to affect the nervous system (Porte et al., 2002; Banks et al., 2012). The knockdown of insulin receptors in the hypothalamus of rats triggers depressive behaviors (Kleinridders et al., 2015). Consistently, insulin administration improves working memory in both human and animal studies (Gupta et al., 2014a; Zhao et al., 2019), confirming that insulin is involved in hippocampal neurogenesis linked with depression.

Considering that insulin resistance is a shared metabolic abnormality among many individuals with DM and depression, repurposing therapies approved for treating insulin resistance might be useful in the treatment of comorbid DM and depression (Hamer et al., 2019). A clinical study of 488 patients with major depression found that selective agonists of the nuclear transcription factor peroxisome proliferator-activated receptor-γ (PPAR-γ), which are used for the treatment of insulin resistance-related diabetes, exhibit significant antidepressant properties by virtue of their ability to ameliorate insulin resistance and glucose tolerance (Colle et al., 2017). Further, the classic antidiabetic drug metformin promotes anxiolytic and antidepressant-like responses in insulin-resistant mice (Zemdegs et al., 2019), suggesting that the management of depression-associated diabetes through targeting insulin resistance is effective and practicable.

### TCM and Natural Products for Treating Insulin Resistance Associated with Comorbid Depression and Diabetes

Decoction of Pinellia and Magnolia Bark (Banxia-Houpu decoction) is a well-known formula of TCM consisting of *Pinellia ternata *(Thunb.) Makino*, Magnolia officinalis *Rehd. et Wils.** *, Smilax glabra* Roxb.*, Perilla frutescens* (L.) Britt., and *Zingiber officinale *Rosc*.* In chronic unpredictable mild stress (CUMS) rats, decoction of Pinellia and Magnolia Bark has been found to regulate InsR/IRS1/AKT insulin signaling, to decrease the level of serum corticosterone (CORT), and to improve glucose tolerance in both peripheral liver and brain regions (Jia et al., 2017). Clinical trials have showed that Ease Powder of Moutan Bark and Cape Jasmine Fruit (Danzhi Xiaoyao powder, DZXYP) has the ability to regulate glucose and improve the symptoms of depression and insomnia (Zhang and Xiao, 2008). Likewise, a meta-analysis of DZXYP for type 2 DM complicating depression suggested that treatment with DZXYP improved fasting blood glucose, HbA1c, Hamilton depression scale score, and self-rating depression scale score (Zhang et al., 2017). Mechanism research revealed that DZXYP alleviates diabetes-associated depression through the IRS2-PI3K signaling pathway in the liver tissues of rats (Wang et al., 2013). Two other studies verified the hypoglycemic and antidepressant effects of Glucose-Lowering and Relieving Depression Decoction for Tonifying the Kidney-yin (Zuogui Jiangtang Jieyu prescription, ZGJTJY) (Wang et al., 2014; Yang et al., 2020), showing that ZGJTJY can effectively regulate the insulin signaling pathway and improve insulin resistance in the hippocampus of rats with diabetes-related depression (Yang et al., 2020). In line with these observations, the hydroalcoholic extract of *Urtica dioica* L. leaves was found to reverse the decline in hippocampal GLUT4 expression and plasma CORT induced by dexamethasone, which correlated with its ability to improve hyperglycemia and associated depressive-like behavior (Patel and Udayabanu, 2014) (Tables 1, 2; Figure 1).

A large number of monomeric compounds possess hypoglycemic and antidepressant properties via different mechanisms. Among them, curcumin and quercetin are representative purified compounds capable of improving insulin resistance. Curcumin has been reported to enhance insulin sensitivity and increase the hepatic glycogen content by upregulating the phosphorylation of IRS-1 and AKT and inhibiting GSK-3*β* in rats with depression complicated by insulin resistance using a 12-week exposure to chronic mild stress, which were conducive to reversing the metabolic abnormalities and depressive-like behaviors (Shen et al., 2017). In the Porsolt forced swimming-induced behavioral despair test, quercetin treatment was found to reduce the immobility period and to reverse depressive-like behavior in a dose-dependent manner in streptozotocin (STZ)-induced diabetic mice (Anjaneyulu et al., 2003). Further study has indicated that quercetin administration in male Swiss albino mice can significantly attenuate insulin resistance and elevate hippocampal GLUT4 levels (Mehta et al., 2017). Similarly, umbelliferone treatment (20 and 40 mg/kg, for 3 weeks) in CUMS-induced rats effectively improved depression symptoms and maintained blood glucose balance. Western blot analysis demonstrated that umbelliferone administration markedly increases the phosphorylation of InsR, IRS-1, AKT, PI3K, and GSK-3*β*, suggesting an ameliorative effect of umbelliferone against insulin resistance (Su et al., 2016) (Table 3; Figure 1).

## TCM and Natural Products Linked with Oxidative Stress and Inflammation

### The Role of Oxidative Stress and Inflammation in Comorbid Depression with DM

Oxidative stress and inflammation interact in the pathophysiology of both DM and depression. For instance, the elevated release of ROS, a characteristic of oxidative stress, not only causes direct damage to the insulin-producing pancreatic *β*-cells but also results in inflammatory processes, immune activation, increased oxidation of monoaminergic neurotransmitters, and lipid peroxidation, all of which correlate with DM and depression (Gerber and Rutter, 2017; Forrester et al., 2018; Reus et al., 2019). Similarly, increased concentrations of cytokines resulting from inflammatory processes also lead to the apoptosis of pancreatic *β*-cells and insulin resistance as well as enhanced oxidative stress in the brain and activation of the HPA axis and the tryptophan-kynurenine pathway, contributing to the risk of coexistence of diabetes and depression symptoms (Moulton et al., 2015). There is evidence that patients with diabetes or depression have significantly increased levels of oxidative stress markers including malondialdehyde (MDA), F2-isoprostanes, and 8-OH 2-deoxyguanosine (8-OHdG), as well as proinflammatory cytokines, such as interleukin-1 (IL-1), interleukin-6 (IL-6), and tumor necrosis factor-α (TNF-α). In addition, decrease in the content or activity of important antioxidant enzymes, such as superoxide dismutase (SOD) and catalase (CAT), has also been observed (Myint et al., 2005; Ballak et al., 2015; Lindqvist et al., 2017; Luc et al., 2019).

Moreover, injection of cytokines in animals and humans can induce depression-like symptoms and diabetes (Horikawa et al., 2003; Sauter et al., 2015; Abdul Aziz et al., 2016). Consistent with these findings, clinical trials have indicated that nonsteroidal anti-inflammatory drugs (NSAIDs) and cytokine inhibitors may yield antidepressant effects (Kohler et al., 2016). Treatment with antioxidants including N-acetylcysteine and deferoxamine ameliorates diabetes-induced depressive-like behavior (Reus et al., 2016). Thus, blockade of oxidative stress and targeting the proinflammatory signaling pathway might be potential therapeutic strategies for comorbid depression in diabetes.

### TCM and Natural Products with Antioxidative Stress and Inflammation Effects against Comorbid Depression and Diabetes

The antioxidative stress and anti-inflammatory activities of TCM and natural extracts and compounds have been widely explored. In this sense, decoction of Pinellia and Magnolia Bark was found to inhibit NLRP3 inflammasome activation in a number of tissues, such as the liver, hypothalamus, hippocampus, and prefrontal cortex, which contributes to the prevention of hyperglycemia and depressive-like behavior in CUMS rats (Jia et al., 2017). The ZGJTJY treatment mentioned above was found to reduce IL-1*β* and TNF-α levels in the hippocampus in the rat model of diabetes complicated by depression induced by high-fat emulsion, injection of STZ via coccygeal vein, and chronic stress (Yang et al., 2018). Inspired by a meta-analysis showing the effectiveness and safety of Bupleurum Powder for Relieving Liver-qi (Chaihu-Shugan San, CHSGS) in the clinical treatment of depression (Wang et al., 2012b), a recent study by Jia et al. further confirmed that CHSGS improves glucose tolerance in the rat model of depression. Briefly, this formula was found to improve insulin signaling and to suppress both the TLR4/MyD88/NF-κB pathway and the activation of NLRP3 inflammasome, thereby reducing blood glucose levels and ameliorating depressive-like behaviors through inhibiting liver-brain inflammation axis (Jia et al., 2018). The previous study established a relatively precise effect of the Chinese Angelica Decoction for Replenishing Blood (Danggui-Buxue decoction, DBD) on DM with depression (Wang et al., 2018), and investigation of the mechanisms showed that DBD reduces serum levels of inflammatory factors such as TNF-α, CRP, IL-6, and IL-8, thereby inhibiting the systemic inflammatory response and achieving the positive regulation of DM complicated with depression (Wang et al., 2020a) (Table 1; Figure 1).

In the same way, hydroalcoholic extracts of *Rosa canina* L. fruit, an Iranian traditional medicinal herb with antioxidant activities, were found to enhance the attenuation of depressive-like behavior and recognition memory impairment through regulation of the oxidative stress marker MDA and total antioxidant capacity (TAC) in diabetic mice (Sadigh-Eteghad et al., 2011; Farajpour et al., 2017). Likewise, *Aloe vera* gel was found to increase activities of hippocampal SOD and CAT and to reduce MDA levels in the hippocampus tissue of STZ-induced diabetic rats, thus improving oxidative status and behavioral deficits (Tabatabaei et al., 2017). In STZ-induced diabetic rats, garlic (*Allium sativum* L.) (0.5 g/kg, gavage, 10 days) treatment also decreased the total duration of immobility, attenuated MDA levels, and increased SOD and glutathione peroxidase (GSH-Px) activities, indicating that garlic alleviates depression-related behaviors partly by attenuating brain oxidative stress (Rahmani et al., 2020). Additionally, extracts of *Urtica dioica* L. were proven to be effective for anxiety- and depressive-like behavior mediated by DM via decreasing the expression of TNF-α in the hippocampal area (Patel et al., 2018) (Table 2; Figure 1).

Numerous phytochemical molecules with various pharmacological effects exhibit antidepressant activity and improve glucose tolerance through the modulation of oxidative stress and/or inflammatory response. For instance, the anxiolytic-like and neuroprotective effects of resveratrol, a stilbenoid isolated from multiple medicinal herbs, correlate with its ability to strengthen the antioxidant action of SOD enzymes and to attenuate the expression of TNF-α, IL-6, and NF-κB in the hippocampus of diabetic rats (Jing et al., 2013; Sahin et al., 2019). Two phenolic compounds, ellagic acid and protocatechuic acid, were able to suppress inflammatory biomarkers and ameliorate diabetes-induced cognitive deficits reflected by the improvement of locomotor activity (Adedara et al., 2019; Farbood et al., 2019). In diabetic db/db mice, gastrodin administration and treatment with ginsenoside compound K were found to significantly attenuate blood glucose levels and to reverse behavioral impairment and cognitive dysfunction due to the inhibitory effects of these compounds on endoplasmic reticulum (ER) stress and NLRP3 inflammasome activation in the hippocampus (Ye et al., 2018; Li et al., 2020). Similar outcomes also have been observed for several molecules from medicinal and edible plants. Naveen Shivaved et al. revealed that ascorbic acid, which is abundant in fresh fruits and vegetables, ameliorates oxidative stress and inflammatory response by reducing catalase levels and increasing SOD content, CAT activity, and IL-10 levels in the prefrontal cortex of diabetic rats, indicating the therapeutic potential of ascorbic acid against diabetes comorbid depression (Shivavedi et al., 2019). In addition, studies by Zhou et al. showed that carotenoids, including astaxanthin and zeaxanthin, downregulate IL-6 and IL-1*β* in the hippocampus and protect neurons from hyperglycemic damage, implicating that depression can be prevented by astaxanthin and zeaxanthin through the inhibition of hippocampal inflammation in diabetic mice (Zhou et al., 2017; Zhou et al., 2018). Finally, *β*-caryophyllene, a natural sesquiterpene found in some food condiments, was proved to attenuate the expression of cytokines and depressive-like behavior in experimental diabetic mice subjected to the marble test, forced swim test, and tail suspension test (Aguilar-Avila et al., 2019) (Table 3; Figure 1).

## TCM and Natural Products with Effects on Changes in Nervous System

### Nervous System Changes and Comorbid Depression with Diabetes

In patients with DM and depressive disorders, a wide variety of disturbances may affect the central nervous systems, including the overactivation of the HPA axis, decreased monoamine neurotransmitters, and dysfunctional brain-derived neurotrophic factor (BDNF) (Donato, 2012; Biessels and Reijmer, 2014). Firstly, patients with diabetic depression present significant HPA axis dysfunction as well as higher levels of cortisol than control patients (Joseph and Golden, 2017). Hyperactivity of the HPA axis results in elevated levels of cortisol, which impairs the ability of insulin to transfer the intracellular glucose transporter GLUT4 to cell membrane and hampers neurogenesis in the hippocampus (Coderre et al., 1996; Moulton et al., 2015; Shirazi et al., 2015). Therefore, the chronic exposure to excess cortisol eventually leads to insulin resistance and concomitant metabolic syndromes including type 2 diabetes as well as mental disorders including depression (Rhyu et al., 2019). Furthermore, the decreased or insufficient secretion of monoamine transmitters in the brain, such as norepinephrine (NE), dopamine (DA), γ-aminobutyric (GABA), and serotonin (5-HT), is also a major cause of comorbid diabetic depression (Hashim et al., 2016; Ma et al., 2019; Mukherjee and Chaturvedi, 2019; Zhang et al., 2020). Experimental studies have revealed that impairment of 5-HT causes nerve cell damage and decreases neurogenesis, leading to anxiety and depression (Mahar et al., 2014; Yohn et al., 2017). In addition to acting as a neurotransmitter that regulates the function of the central nervous system, 5-HT also exerts multiple physiological functions in the periphery, and several studies have revealed the relationship between the peripheral 5-HT system and some diseases including type 2 diabetes (Fu et al., 2018). Two studies by Gupta et al. showed the counteractive effect of 5HT_3_ receptor antagonists against diabetes-induced depressive phenotypes in STZ-induced diabetic mice, further revealing the implication of the serotonergic system in diabetic depression (Gupta et al., 2014b; Gupta et al., 2016). As a neurotrophic factor, BDNF promotes the proliferation, remodeling, and regeneration of nerve cells and thus regulates neuroplasticity. Serum BDNF concentrations have been found to be decreased in patients with depression and diabetes (Karege et al., 2005; Krabbe et al., 2007). Convincing evidence supports the view that BDNF plays an important role in the pathogenesis of depression and diabetes.

Briefly, BDNF may improve glucose metabolism and reduce food consumption by modulating the production of insulin, leptin, ghrelin, neurotransmitters/neuropeptides, and proinflammatory cytokines associated with energy homeostasis. Clinical studies have shown that depressive states could be reversed by administration of BDNF (Wang et al., 2012a). Moreover, BDNF is positively regulated by the ER chaperon sigma-1 receptor (S1R), and studies have shown that fluvoxamine, a S1R agonist, ameliorates depression-like behaviors in DM-induced depression (Lenart et al., 2016). In short, the repair of the nervous system serves as a therapeutic regimen for the prevention of DM-associated depression.

### TCM and Natural Products Targeting Nervous System Changes Associated with Comorbid Depression and Diabetes

As mentioned above, changes in the nervous system are critical for the development of diabetic depression. From this perspective, Decoction for Tonifying Kidney and Relieving Depression (Yishen Jieyu decoction) improves HPA axis function and decreases the cortisol level in patients with DM-related depression and TCM syndrome, which is diagnostic of kidney deficiency and liver-qi stagnation (Lu, 2012). HPA axis disorder also causes an abnormal increase in CORT in rodents, which is closely related to two essential proteins expressed in the hippocampus, 11*β*-hydroxysteroid dehydrogenase type 1 (11*β*-HSD1), and glucocorticoids (GR) (Wang et al., 2015; Meijer et al., 2018). The aforementioned ZGJTJY (10.26 g/kg) also increases motor activities and improves cognitive ability in rats with diabetes-related depression by increasing the expression of GR and decreasing 11*β*-HSD1 (Wang et al., 2015; Yang et al., 2019). Taking into consideration the observed effectiveness of Formula for Soothing Liver and Tonifying Spleen (Shugan Jianpi formula, SGJP) in the treatment of diabetes-related depression (Wang, 2008; Li, 2017), a recent study by Lei et al. indicated that SGJP improves depressive symptoms in CUMS and STZ-induced rats by upregulating the expression of BDNF mRNA and protein (Lei et al., 2017). Similarly, BDNF elevation is yet another mechanism of DBD against comorbid depression and diabetes. In CUMS-induced spontaneous diabetic rats, DBD (4.0 g/kg) and its main active ingredient ferulic acid (1.36 mg/kg) could increase serum and hippocampal BDNF levels to improve depression-like behavior, an effect that might relate to the activation of the CREB/TrkB signaling pathway (Wang et al., 2020b) (Table 1; Figure 1).

Likewise, the methanolic extract of *Brassica juncea* leaves (100, 200, and 400 mg/kg/day, p.o.) has been found to display antidepressant activity in alloxan monohydrate-induced diabetic rodents and to compensate for the low levels of DA, NE, and 5-HT in the brain of diabetic rats (Thakur et al., 2014). Cannabidiol (CBD), a non-psychotomimetic compound derived from *Cannabis sativa*, has been identified as a promising small molecule for the treatment of psychiatric disorders. It is reported that CBD might be effective in the treatment of diabetic depression, and this effect seems to be mediated by the activation of the serotonergic system through the 5-HT_1A_ receptor (de Morais et al., 2018; Jesus et al., 2019). In the diabetic rat model induced by high-fat diet and low-dose STZ, treatment of *Morus alba* L. and the combination of *Pueraria lobata (Willd.) Ohwi* and *Crataegus pinnatifida Bunge* could reduce blood glucose levels and attenuate depressive-like behaviors, which might be partially ascribed to the upregulated BDNF expression in the prefrontal cortex (Luo et al., 2016; Ye et al., 2017). In addition, restoration of BDNF is also involved in the geniposide- and ellagic acid-mediated alleviation of depression-like behavior in STZ-evoked diabetic mice (Wang et al., 2016; Farbood et al., 2019) (Tables 2, 3; Figure 1).

## Conclusion

Recently, some hospital-based cross-sectional studies have further clarified the comorbidity between DM and depression (Khassawneh et al., 2020; Tusa et al., 2020). In this review, we summarized TCM formulae and natural products with antidiabetic depression activities as well as several mechanisms related to their activities. Although the TCM and natural bioactive products discussed here greatly expand the spectrum of potential candidates for the prevention and treatment of the comorbidity of depression with DM, their efficacy should be further evaluated in randomized, double-blinded, and placebo-controlled clinical trials. Moreover, the active components in TCM or natural extracts, such as ZGJTJY and hydroalcoholic extract of *Urtica dioica* leaves, remain unclear in the literature. More importantly, the underlying mechanisms in most studies were only partially understood and mainly focused on changes in signaling pathways (Jia et al., 2018), cytokines and neurotransmitters content (Thakur et al., 2014; Aguilar-Avila et al., 2019; Farbood et al., 2019), and antioxidant enzyme activity (Rahmani et al., 2020). The target(s) with which the bioactive compounds interact and how the changes are triggered should be studied in depth. For example, omics technologies and network pharmacology might be helpful for the identification or prediction of the target of purified compounds or active ingredients in TCM or natural extracts, which may in turn enhance our understanding of the molecular mechanisms underlying the comorbidity between DM and depression. In addition, some studies on natural extracts and purified compounds from medicinal and edible plants suggest the potential of diet or nutritional therapy as an adjuvant for treating depression commonly associated with diabetes (Shen et al., 2017; Shivavedi et al., 2019; Rahmani et al., 2020). Overall, this review provides an important theoretical basis for therapeutic alternatives and potential intervention strategies to ameliorate comorbid DM and depression and broadens the scope of knowledge to guide future studies on this subject.

## Author Contributions

YL, TA, and KM were involved in the development of the subject matter, drafting of the article, design of the figure, critical revision of the article, and final approval of the version to be published; HT and XG were involved in critical revision of the article and final approval of the version to be published; SW and FW were involved in the development of the subject matter, drafting of the article, critical revision of the article, and final approval of the version to be published. All authors have read and approved the final version of the manuscript.

## Funding

This study was supported by the Shandong Provincial Natural Science Foundation (ZR2020QH329, ZR2019BH027 and ZR2019ZD23), Shandong Province Universities’ Development Plan for Youth Innovation Teams (2019-9-202, 2019-201, and 2019KJK013), National Nature Science Foundation of China (81903948), and Shandong Province University Scientific Research Project (J18KZ014).

## Conflict of Interest

The authors declare that the research was conducted in the absence of any commercial or financial relationships that could be construed as a potential conflict of interest.

## Glossary

11*β*-HSD1: 11*β*-hydroxysteroid dehydrogenase type 1
5-HT: serotonin
8-OHdG: 8-OH 2-deoxyguanosine
BDNF: brain-derived neurotrophic factor
CAT: catalase
NSAIDs: nonsteroidal anti-inflammatory drugs
CBD: cannabidiol
CHSGS: Chaihu-Shugan San or Bupleurum Powder for Relieving Liver-qi
CORT: corticosterone
CUMS: chronic unpredictable mild stress;
DA: dopamine
DBD: Danggui-Buxue decoction or Chinese Angelica Decoction for Replenishing Blood
DM: diabetes mellitus
DZXYP: Danzhi Xiaoyao powder or Ease Powder of Moutan Bark and Cape Jasmine Fruit
ER: endoplasmic reticulum
GLUT: glucose transporter
GR: glucocorticoid
GSH-Px: glutathione peroxidase
GSK: glycogen synthase kinase
HFD: high-fat diet
HPA: hypothalamic-pituitary-adrenal
IDF: International Diabetes Federation
IL-1: interleukin-1
IL-6: interleukin-6
InsR: insulin receptor
IRS: insulin receptor substrates
MDA: malondialdehyde
NE: norepinephrine
PPAR-γ: peroxisome proliferator-activated receptor-gamma;
S1R: sigma-1 receptor
SGJP: Shugan Jianpi formula or Formula for Soothing Liver and Tonifying Spleen
SOD: superoxide dismutase
SREBP1-c: sterol regulatory element-binding protein-1c
STZ: streptozotocin
TAC: total antioxidant capacity
TCM: traditional Chinese medicine
TNF-α: tumor necrosis factor-alpha
WHO: World Health Organization
ZGJTJY: Zuogui Jiangtang Jieyu prescription
